# Varying Dietary Component Ratios and Lingonberry Supplementation May Affect the Hippocampal Structure of ApoE–/– Mice

**DOI:** 10.3389/fnut.2022.565051

**Published:** 2022-02-16

**Authors:** Dmytro Shepilov, Tatiana Kovalenko, Iryna Osadchenko, Kateryna Smozhanyk, Nittaya Marungruang, Galyna Ushakova, Diana Muraviova, Frida Hållenius, Olena Prykhodko, Galyna Skibo

**Affiliations:** ^1^Department of Cytology, Bogomoletz Institute of Physiology, Kyiv, Ukraine; ^2^Department of Food Technology, Engineering and Nutrition, Lund University, Lund, Sweden; ^3^Department of Biochemistry and Physiology, Oles Honchar Dnipro National University, Dnipro, Ukraine

**Keywords:** hippocampus, lingonberries, dietary fiber, glial cells, ApoE–/– mice, low- and high-fat diets, starch, structural synaptic plasticity

## Abstract

**Objective:**

This study aimed to investigate and compare the morphological and biochemical characteristics of the hippocampus and the spatial memory of young adult ApoE–/– mice on a standard chow diet, a low-fat diet (LFD), a high-fat diet (HFD), and an HFD supplemented with lingonberries.

**Methods:**

Eight-week-old ApoE–/– males were divided into five groups fed standard chow (*Control*), an LFD (*LF*), an HFD (*HF*), and an HFD supplemented with whole lingonberries (*HF*+*WhLB*) or the insoluble fraction of lingonberries (*HF*+*InsLB*) for 8 weeks. The hippocampal cellular structure was evaluated using light microscopy and immunohistochemistry; biochemical analysis and T-maze test were also performed. Structural synaptic plasticity was assessed using electron microscopy.

**Results:**

ApoE–/– mice fed an LFD expressed a reduction in the number of intact CA1 pyramidal neurons compared with HF+InsLB animals and the 1.6–3.8-fold higher density of hyperchromic (damaged) hippocampal neurons relative to other groups. The LF group had also morphological and biochemical indications of astrogliosis. Meanwhile, both LFD- and HFD-fed mice demonstrated moderate microglial activation and a decline in synaptic density. The consumption of lingonberry supplements significantly reduced the microglia cell area, elevated the total number of synapses and multiple synapses, and increased postsynaptic density length in the hippocampus of ApoE–/– mice, as compared to an LFD and an HFD without lingonberries.

**Conclusion:**

Our results suggest that, in contrast to the inclusion of fats in a diet, increased starch amount (an LFD) and reduction of dietary fiber (an LFD/HFD) might be unfavorable for the hippocampal structure of young adult (16-week-old) male ApoE–/– mice. Lingonberries and their insoluble fraction seem to provide a neuroprotective effect on altered synaptic plasticity in ApoE–/– animals. Observed morphological changes in the hippocampus did not result in notable spatial memory decline.

## Introduction

Apolipoprotein E (ApoE) is a protein that binds and transports cholesterol throughout the body, particularly in the CNS and liver (1, 2). The protein plays a crucial role in maintaining neuronal functions, acting as a trophic, antioxidant factor and a mediator of the immune response during brain development and in response to cerebral damage (3). Additionally, ApoE is involved in debris clearance from brain tissues and the stimulation of nerve cell regeneration under normal and some pathological states, including aging and neurodegenerative diseases (4).

The ApoE-knockout mouse model (ApoE–/–) is widely used to study the consequences of ApoE deficiency (5). ApoE–/– mice demonstrate lipid metabolism deviations, such as hypercholesterolemia, and elicit atherosclerotic lesion formation with further inflammation and degradation of the extracellular matrix, especially with age (6). Reportedly, the plasma cholesterol level in ApoE–/– mice is 5- to 10-fold higher than in wild-type mice on a high-fat diet (HFD) (7). There is also evidence that ApoE–/– mice have abnormal tau protein phosphorylation, cholinergic dysfunction, synaptic loss, as well as memory and antioxidant metabolism impairments, which may negatively impact the function and integrity of the nervous system (8, 9).

The loss of synaptophysin-positive nerve terminals and MAP2-positive dendrites in the neocortex and hippocampus of ApoE-deficient mice on a standard diet has been detected using immunohistochemistry. There have also been reports on a drastic decrease in α- and β-tubulin immunoreactivity in ApoE–/– mice that might indicate impaired intracellular transport. In addition, ApoE-deficient mice tend to have an increased activation level of microglia (10). ApoE–/– mice, after HFD feeding, have been shown to exhibit cell swelling, nuclear hyperchromatosis, and karyopiknosis of the pyramidal neurons, as well as lipid accumulation in the hippocampus. Furthermore, the increased expression of several proteins linked to apoptosis activation (PCSK9, BACE1, caspase-3, and Bax) has been observed in CA3 neurons (11). Another study revealed that ApoE deficiency enhances the risk of hippocampal oxidative damage, while an HFD deteriorates oxidation (12). Also, high-cholesterol diets affect microglia by increasing the formation of inflammatory mediators (13). In contrast, caloric restriction in ApoE–/– mice initiates neuroprotection manifested through the growing number of PSD95-positive neurons and better performance in Morris water maze (14), a learning and spatial memory test that has been shown to strongly correlate with hippocampal synaptic plasticity and function (15). Yet, some researchers have emphasized that a neuroinflammatory response with increased CD68+ cells in the CA1 area of ApoE–/– mice arises even on a standard rodent chow diet, and the level of inflammation remains unchanged when switching to an HFD (4).

Recently, the neuroprotective effects of berries have garnered increasing attention due to the reported antioxidant and anti-inflammatory properties derived from their bioactive components, such as dietary fiber and polyphenols (16). An extract of lingonberries, *Vaccinium vitis-idaea*, rich in dietary fiber and polyphenols, has been demonstrated to have a beneficial effect on cultured *in vitro* cardiomyocytes, neurons, and astrocytes (17, 18). Our prior study in 8-week-old ApoE–/– mice showed that the inclusion of lingonberries or their insoluble fraction in an HFD (38% of kcal from fat) feed for 8 weeks modified gut microbiota composition, which was associated with improvements in metabolic parameters, short-chain fatty acid profiles in serum, and increased hippocampal synaptic density (19).

To extend that study, the present research further explores the morphological and biochemical parameters of the hippocampus of young adult ApoE–/– mice after 8 weeks of feeding them an HFD containing whole lingonberries (HF+WhLB) or the insoluble fraction of lingonberries (HF+InsLB). We also investigated the cognitive function and hippocampal CA1 area morphology of ApoE-deficient mice on a standard chow, the low-fat diet (LFD), and an HFD. Our results may provide a better understanding of the effects of different levels of fats and other constituents in a diet on the structure of the hippocampus of ApoE–/– mice and lend further support to the potential benefit of bioactive components from berries, specifically lingonberries, on the brain.

## Materials and Methods

### Animals and Diets

**Figure 1:**
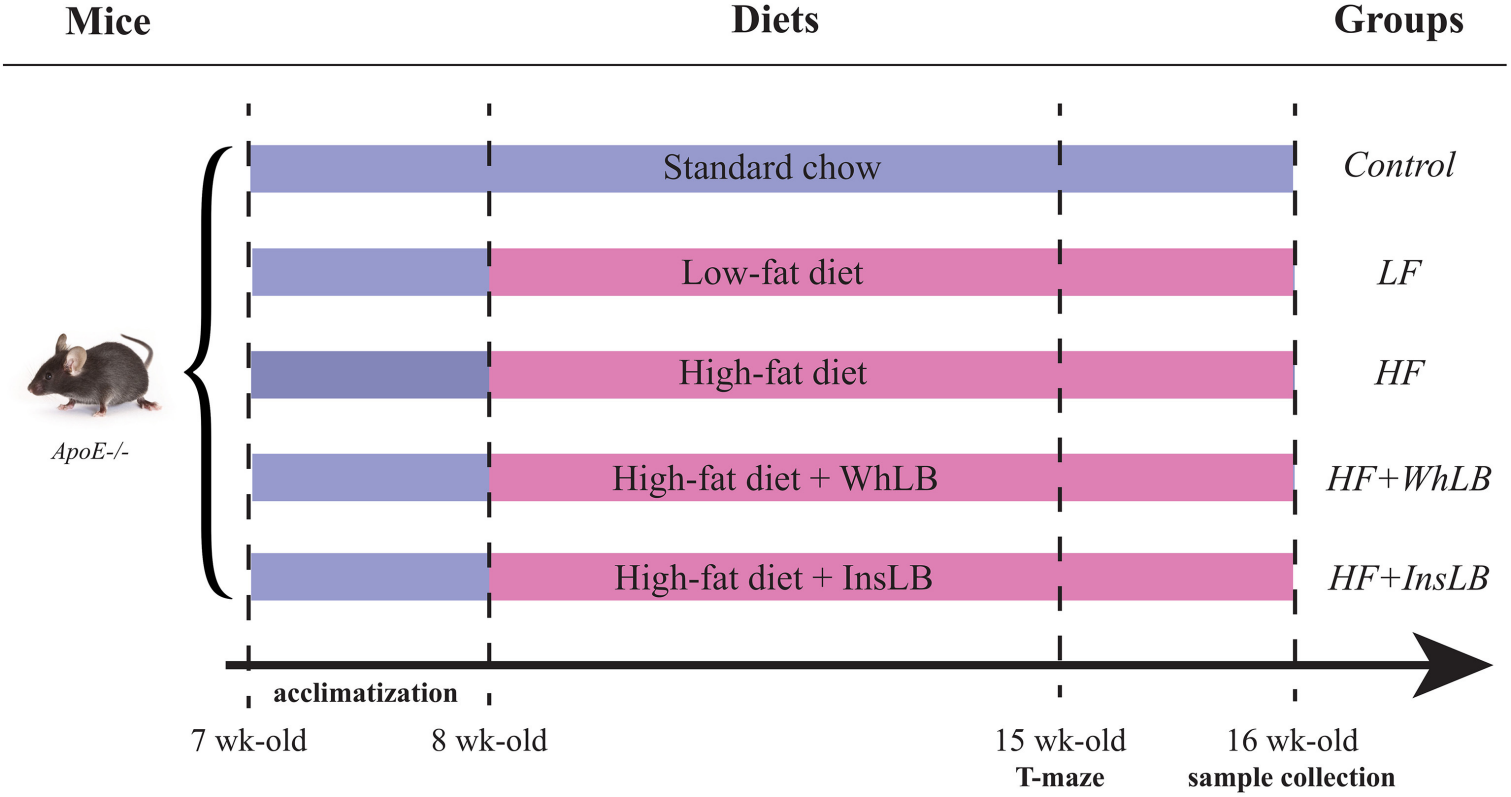
Schematic representation of the experiment. Male ApoE–/– mice were acquired at the age of 7 weeks and left to adapt to the environment and diets for a week. Animals were fed with the different diets for 8 weeks: a standard chow, a low-fat diet, and a high-fat diet (either alone or with whole lingonberries and their insoluble fraction) before perfusion and sample collection.

Male ApoE–/– mice (*n* = 49; Taconic Biosciences Inc., Denmark) arrived at the animal facility at the age of 7 weeks (21.9 ± 0.2 g) and were left to adapt to the environment (22°C, 12h light-dark cycle) and diets for 1 week prior to starting the experiment. Following the adaptation period, animals were randomly allocated to 5 groups (*n* = 9–10/group) based on mouse strain and nutrition: (1) control—ApoE–/– mice fed a standard rodent chow diet (7.4% of kcal from fat, containing soybean oil) (RM1, SDS, UK); (2) LF ApoE–/– mice fed a customized LFD (12% of kcal from fat, containing soybean oil) (Research Diets Inc., New Brunswick, USA); (3) HF ApoE–/– mice fed a customized HFD (38% of kcal from fat, containing soybean oil, lard, and cholesterol) (Research Diets Inc., New Brunswick, USA); (4, 5) HF+WhLB and HF+InsLB ApoE–/– mice fed a customized HFD with the inclusion of whole lingonberries or their water-insoluble fraction, respectively (Research Diets Inc., New Brunswick, USA) (Figure 1). Information about the diet composition is given in Table 1. A more detailed description of the diet preparation and inclusion of the berries is provided elsewhere (19). The experimental period lasted 8 weeks, at the end of which all the animals were 16 weeks old (commonly referred to as young adults). Duration of the feeding is based on our prior research on the bioactive effects of lingonberries (20). The investigation was carried out according to the principles in Directive 2010/63/EU of the European Union and approved by the local ethical committee at Lund University (Sweden).

**Table 1:** Composition of the experimental diets.

	**LF**		**HF**		**HF + WhLB**		**HF + InsLB**	
**Nutrient**	**gm%**	**kcal%**	**gm%**	**kcal%**	**gm%**	**kcal%**	**gm%**	**kcal%**
Protein	15	16	15	14	15	14	15	14
Carbohydrate	72	72	57	48	39	34	43	37
Fat	5	12	18	38	18	38	18	38
**Ingredient**	**gm**	**kcal**	**gm**	**kcal**	**gm**	**kcal**	**gm**	**kcal**
Casein	157.5	630	157.5	630	157.5	630	157.5	630
Corn starch	713.5	2,540	544.9	1,940	362.9	1,292	401.7	1,430
Sucrose	50	200	50	200	50	200	50	200
Cellulose	60	0	60	0	0	0	0	0
Lingonberries (whole berries)^a^	0	0	0	0	264.3	648^c^	0	0
Lingonberries (insoluble fraction)^b^	0	0	0	0	0	0	215.3	510^c^
Soybean oil	50	450	50	450	50	450	50	450
Lard	0	0	140	1,260	140	1,260	140	1,260
Cholesterol	0	0	10	0	10	0	10	0
Mineral mix	35	0	35	0	35	0	35	0
Vitamin mix	10	40	10	40	10	40	10	40
**Total**	**1,076**	**3,860**	**1,057.4**	**4,520**	**1,079.7**	**4,520**	**1,069.5**	**4,520**

a *60 g/kg (dwb) total dietary fiber*.

b *60 g/kg (dwb) insoluble dietary fiber*.

c *Calculated energy from carbohydrate content, excluding dietary fiber content in lingonberries or lingonberry insoluble fraction*.

### T-Maze Spontaneous Alternation Test

The spatial memory of all mice was evaluated using the T-maze spontaneous alternation test 1 week before the end of the feeding period, i.e., after 7 weeks on the diets. The test was carried out per Deacon and Rawlins, with some modifications (21). Briefly, each mouse was carefully placed at the starting arm of the maze for 10 s before the animal was left to spontaneously choose one of the two arms (left or right; no rewarding system). Once the mouse chose the arm, the guillotine door was shut for 30 s to allow arm observation. The second trial was performed within 1 min of the first; the mouse was returned to the starting point and allowed to choose one of the two arms again spontaneously. Alternation, i.e., choosing the arm that was not visited before, was expected in the second trial. Three sets of trials a day (morning, afternoon, and evening hours) were performed for each mouse to avoid the influence of the daily cycle (19). The percentage of alternations and latency to choose (time spent to choose the arm) in the second trial were calculated.

### Sample Collection

For morphological studies, 3 mice/group were anesthetized with isoflurane inhalation (Abbott Scandinavia AB, Sweden) and perfused transcardially with 0.1 M phosphate buffer + 0.3% heparin (+37°C), followed by perfusion with a fixative solution (2% paraformaldehyde and 0.25% glutaraldehyde (Sigma-Aldrich, USA) in 0.1 M phosphate buffer, +4°C). After perfusion, the brains were dissected and split into two hemispheres. The left hemispheres were used for immunohistochemistry, whereas the isolated hippocampi from the right hemispheres were examined under light and transmission electron microscopy. Coronal sections of the dorsal hippocampus were obtained from the anatomical areas between 1.65 and 2.48 mm posterior to the bregma, as defined by the Allen Mouse Brain Atlas (**Figures 3A, 4A**) (22). For biochemical assays, the remaining 5–6 animals/group were anesthetized with an overdose of pentobarbital (Nordvace, Sweden), after which their brains were quickly removed; both the left and right hippocampi were isolated on pre-chilled plates and immediately frozen until analysis.

### Immunohistochemistry

Samples for immunohistochemistry were postfixed overnight in the same fixative as above at +4°C. 50-μm-thick sections of the hippocampus were obtained using a vibratome Leica VT1000A (Leica Biosystems, Germany). Free-floating sections were placed in wells of 24-well plates, rinsed with 0.1 M phosphate buffer (pH 7,4), and treated with a blocking solution containing 1% BSA (Sigma-Aldrich, USA) and 0.3% Triton X-100 (Sigma-Aldrich, USA) for 1 h at room temperature. Double immunofluorescent staining was then conducted. Polyclonal chicken antibodies against the glial fibrillary acidic protein (GFAP) (1:1500, Abcam, UK), a specific astrocyte marker, were used for astrocyte detection. Microglial cell identification was carried out with monoclonal rabbit antibodies against Iba-1 (1:750, WAKO, Japan) specifically expressed in microglia. Incubation with primary antibodies lasted 16 h at +4°C. After rinsing, sections were incubated with secondary antibodies; goat anti-chicken conjugated with Alexa Fluor 647 (1:1000, Invitrogen, USA) and donkey anti-rabbit conjugated with Alexa Fluor 488 (1:1000, Invitrogen, USA) for 1.5 h, at room temperature, in the dark. The sections were then rinsed again, placed on histological slides, and mounted with Fluorescence Mounting Media (Dako, Denmark). 6–8 sections per animal and 3–6 images (220 × 220 μm) from each section were analyzed. Images of hippocampal tissues were taken with an FV1000-BX61WI confocal microscope at 40x objective magnification (NA−0.65) (Olympus Corp., Japan).

### Light and Transmission Electron Microscopy

The hippocampi were cut into 400-μm-thick transverse sections with a tissue chopper (McIlwain, UK). These sections were postfixed in 2.5% glutaraldehyde for 1.5 h and 1% OsO_4_ for 1 h (23). Samples were then dehydrated in an ascending series of ethanol followed by dry acetone and embedded in EPON resin (Sigma-Aldrich, Switzerland) per standard protocol (24). The semi-thin (1 μm) sections were produced with an LKB 8800 ultramicrotome (LKB, Sweden), stained with methylene blue, embedded in a Pertex mounting medium (HistoLab Products AB, Sweden), and visualized using an Olympus CX21 light microscope (Olympus Corp., Japan) (objective magnification−20x, NA−0.40). Ten to twelve semi-thin sections per animal and 2–3 images from each section were analyzed.

For electron microscopy, ultra-thin sections (60–70 nm) from the middle part of the CA1 *stratum radiatum* were stained with uranyl acetate and lead citrate and examined using a JEM-100CX transmission electron microscope (Jeol, Japan) at a magnification of ×10,000.

### Morphometric Analysis

ImageJ software, version 1.50i (NIH, USA), was used to study the hippocampal CA1 area structure of mice quantitatively. We counted the average number of intact and hyperchromic neurons per 1 mm length of a pyramidal layer (neuronal density) (25) and measured the density of GFAP-positive and Iba-1-positive cells per 1 mm^2^ of *stratum pyramidale* and *stratum radiatum* and the surface area of astrocytes/microglial cells. The number of synapses and their different types (simple, perforated and multiple types) per 100 μm^2^ of *stratum radiatum* were defined. Simple and perforated synapses were considered synapses with continuous and discontinuous postsynaptic density, respectively. Synapses with more than one spine contacting the same presynaptic terminal were classified as multiple (**Figure 5B**). We also evaluated postsynaptic density (PSD) length.

### Biochemical Analysis (Enzyme-Linked Immunosorbent Assay)

Hippocampal tissues were homogenized in a buffer solution consisting of 25 mM Tris-HCl, 1 mM EDTA, 2 mM β-dithiothreitol, 0.2 mM PMSF, and 0.01% Merthiolate; pH 7,4 (100 mg of tissue/1 ml buffer) (23). Using differential ultracentrifugation, the water-soluble (containing the soluble forms of neural cell adhesion molecule—NCAM and glial fibrillary acidic protein—GFAP), membrane (containing the membrane form of NCAM), and cytoskeletal (containing the filamentous form of GFAP) protein fractions were obtained (26). We employed competitive ELISA to determine astrocyte- and neuron-specific proteins. Polyclonal rabbit primary antibodies against GFAP (Sigma, USA) and NCAM (Abnova, Germany), secondary anti-rabbit-IgG conjugated with HRP (Sigma, USA), and pure standard proteins (GFAP—Boehringer, Germany; NCAM—R&D Systems, USA) were used. Measurements were performed at 492 nm with a spectrophotometer reader, Antos 2010 (Biochrom Ltd., Finland). Protein concentrations were expressed as microgram (μg) per 100 mg of tissue.

### Statistical Analysis

Statistical analysis was performed using GraphPad Prism, version 8.0.2 (GraphPad Software, Inc., USA). Categorical data from behavioral testing (spontaneous alternations) are expressed as percentages and were analyzed using Fisher's exact test. The normality distribution of continuous data was evaluated using the Shapiro-Wilk normality test, and the equality of variances was determined with Levene's test. Continuous behavioral data are given as median values, and interquartile ranges are presented in square brackets. These results are presented graphically as box plots: median values (central lines), interquartile ranges (25th−75th percentile, boxes), minimum and maximum (whiskers). Group comparisons were performed with the Kruskal-Wallis test, followed by Dunn's *post hoc* test. Morphological and biochemical results are presented as the mean ± SEM. Raw data were assessed using one-way ANOVA, followed by Turkey's *post hoc* test. Pearson's correlation analysis was also conducted. R values from 0 to ± 0.29, between ± 0.3 and ± 0.49, and from ± 0.5 to ± 1 were consistent with a low, moderate, and high degree of correlation, respectively. Differences between compared groups were considered statistically significant when *p* < 0.05.

## Results

### T-Maze Spontaneous Alternation

**Figure 2:**
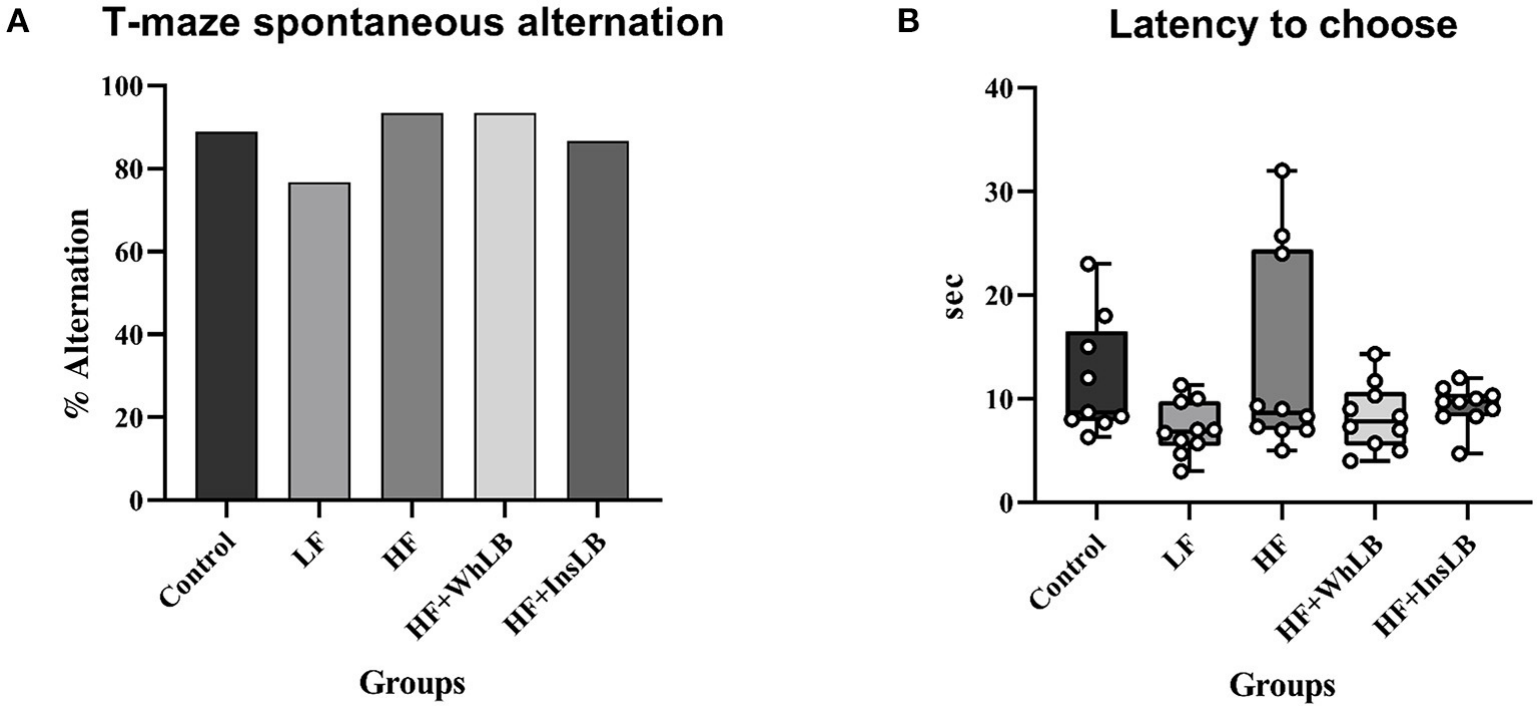
T-maze spontaneous alternation test. **(A)** The percentage of alternation. **(B)** Latency to choose the arm in the second trial. Fisher's exact test was used for categorical data analysis. Continuous data distribution is expressed with the aid of the box and whisker plots: the median is shown by the horizontal line, the box is limited by 25th and 75th percentiles (interquartile range, IQR), while the whiskers represent the full range of data (*n* = 9–10/group). Comparisons among groups were performed with the non-parametric Kruskal-Wallis test, followed by Dunn's *post hoc* test.

LFD-fed mice produced the worst results in the T-maze spontaneous alternation test (76.7% alternation) than animals fed other diets (86.7–93.3%). However, there were no significant differences in the percentage of alternation (an indicator of spatial working memory; Figure 2A) and latency to choose (Figure 2B) between the experimental groups.

### The Density of CA1 Pyramidal Neurons

**Figure 3:**
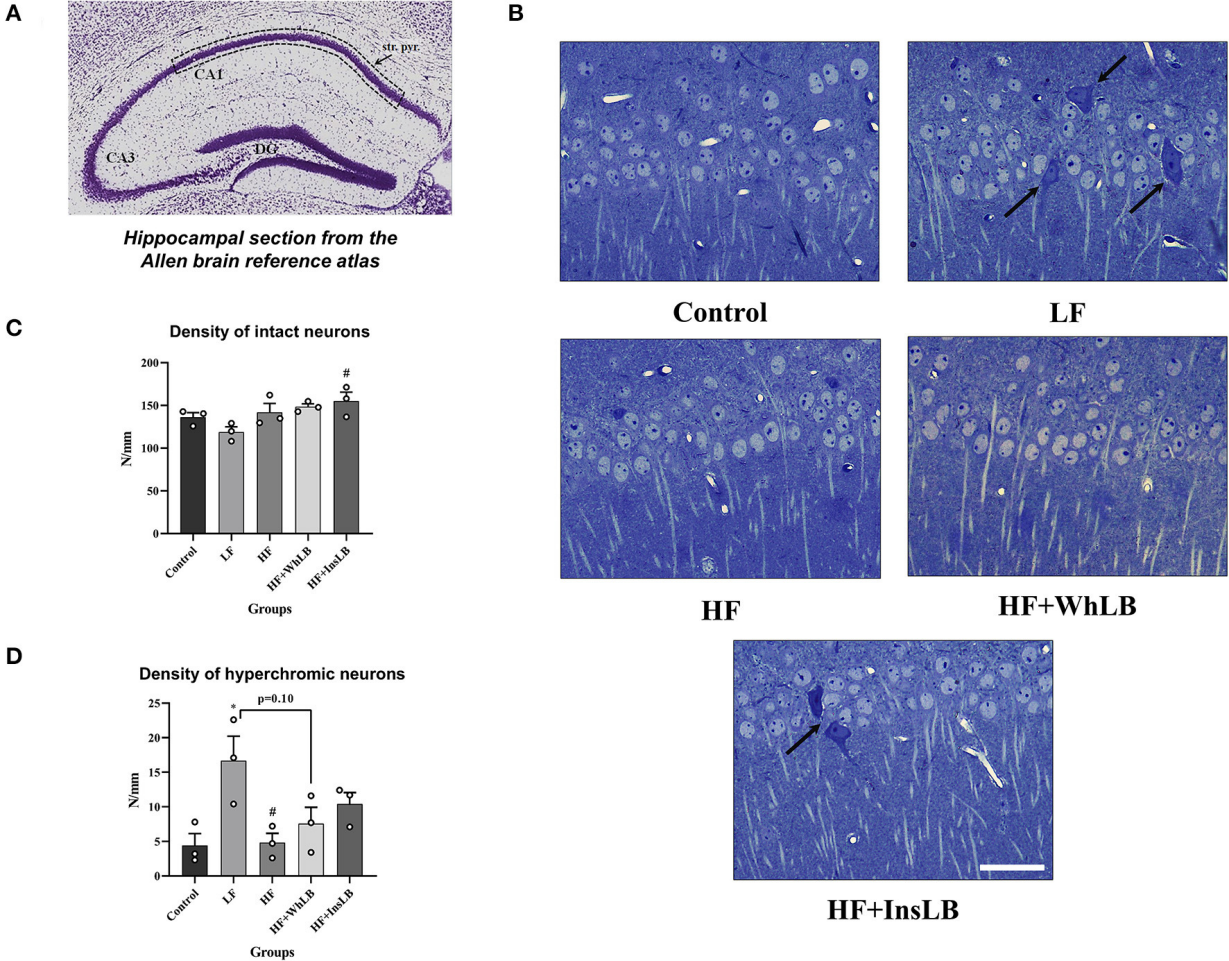
Pyramidal neurons in the hippocampal CA1 area of ApoE–/– mice. **(A)** The reference image of the Nissl-stained coronal section of the dorsal hippocampus obtained from the Allen Mouse Brain Atlas. Dotted lines indicate the area of interest in the *stratum pyramidale*. **(B)** Methylene blue-stained semi-thin sections demonstrate the morphology of a pyramidal layer. Arrows point to hyperchromic damaged cells. **(C)** The density of intact neurons. **(D)** The density of hyperchromic pyramidal cells. Data are expressed as the Mean ± SEM (*n* = 3/group). Comparisons among groups were performed with the one-way ANOVA, followed by Turkey's *post hoc* test. *p < 0.05 indicates significant differences vs. control; ^#^*p* < 0.05 indicates significant differences vs. LF. The scale bar corresponds to 20 μm.

Mice of the control, HF, HF+WhLB, and HF+InsLB groups were characterized by a normal architectonic organization of the hippocampal CA1 area. In those groups, pyramidal neurons had light nuclei with clear borders and visible 1–3 nucleoli, a narrow strip of normochromic cytoplasm around the nucleus, and well-defined descending apical dendrites oriented in the radial direction. Only a few pyramidal cells were hyperchromic. Structural disruptions in the CA1 area, in particular, the hyperchromatosis of the pyramidal cells, and the destruction of several apical dendrites were observed in semi-thin hippocampal sections of LFD-fed animals (Figure 3B). The average density of intact neurons in the control group was 136.3 ± 5.3 cells/mm length of a pyramidal layer. The intact neuronal density in LFD-fed mice was 13, 16, and 20% lower than in the control, HF, and HF+WhLB groups, respectively (no significant differences), and 23% lower vs. the HF+InsLB group (*p* < 0.05) (Figure 3C). Furthermore, there was a significant increase (by 264 ± 16%) in hyperchromic neuronal density in LFD-fed animals, compared to both control (*p* < 0.05) and HFD-fed mice (*p* < 0.05) (Figure 3D).

### Glial Cell Density and Surface Area

**Figure 4:**
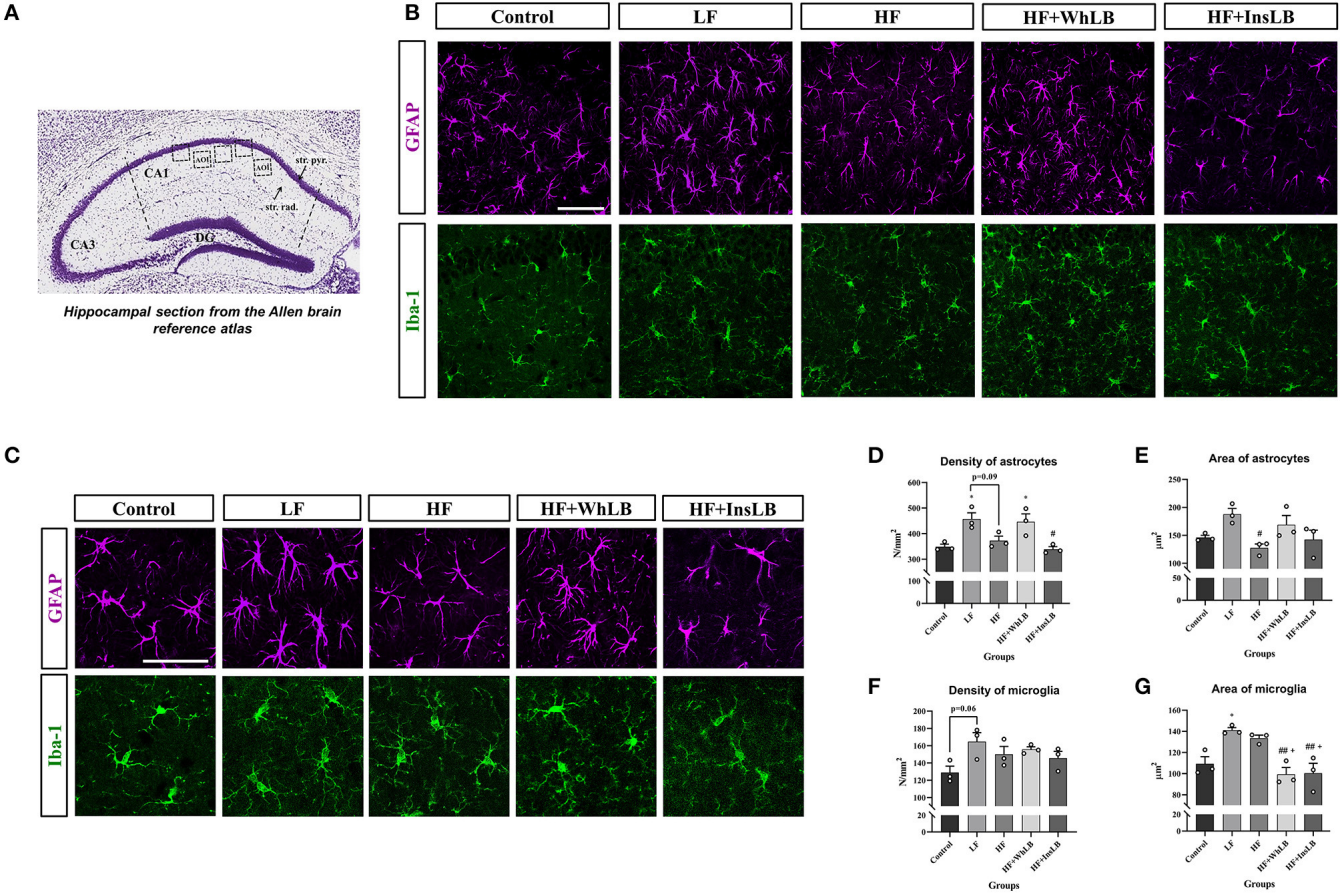
Immunohistochemical analysis of glial cells in the hippocampal CA1 area of ApoE–/– mice. **(A)** The reference image of the Nissl-stained coronal section of the dorsal hippocampus obtained from the Allen Mouse Brain Atlas. Dotted line squares indicate the areas of interest (AOI) in the *stratum pyramidale* and *stratum radiatum*. **(B)** Immunofluorescent-labeled images of GFAP-positive astrocytes and Iba-1-positive microglia at 40x objective magnification, as well as **(C)** cut out and zoomed fragments (crops) from these images for better visualization of the processes and somas of astrocytes and microglial cells. **(D–G)** The morphometric characteristics of glial cells: **(D)** the density of GFAP-positive astrocytes and **(E)** their area; **(F)** the density of Iba-1-positive microglia and **(G)** the area of microglial cells. Data are expressed as the Mean ± SEM (*n* = 3/group). Comparisons among groups were performed with the one-way ANOVA, followed by Turkey's *post hoc* test. *p < 0.05 indicates significant differences vs. control; ^#^*p* < 0.05 and ^##^*p* < 0.01 indicate significant differences vs. LF; ^+^*p* < 0.05 indicates significant differences vs. HF. The scale bar corresponds to 50 μm.

The functional state of glial cells was determined using immunofluorescent microscopy based on their morphological characteristics. It is generally accepted that the reactive state of astrocytes includes the hypertrophy of their cell bodies and an increase in number, thickness, and length of primary processes, as against the resting state (27–29). As illustrated in Figures 4B,C, GFAP-positive cells in both the LF and HF+WhLB groups were characterized by the elongation and multiplication of base processes and the active spreading of side branches compared to other groups. In contrast, soma hypertrophy and the thickening of processes were displayed only in the hippocampi of LFD-fed mice. The mentioned morphological alterations reflected the activation of the astrocyte component in these two groups. LFD-fed ApoE–/– mice showed the highest density of GFAP-positive astrocytes in the hippocampal CA1 area (456.6 ± 24.7 cells/mm^2^ of *stratum pyramidale* and *stratum radiatum*), which was 33 ± 2% more than that of mice on standard chow and an HFD+InsLB (*p* < 0.05) and 23% more than that of mice on an HFD (*p* = 0.09). There was also significant difference (*p* < 0.05) in the number of GFAP-positive cells between HF+WhLB-fed animals and their standard chow-fed counterparts (Figure 4D). As in the case of cell density, the astrocyte area increased in the LF group compared to the HF (*p* < 0.05) one (Figure 4E).

Microglial cells with small and rounded somas and thin, long, and highly branched processes were considered resting microglia. An enlargement of soma with a shape change (cell body becomes bi- or tripolar, ellipsoid or rod-shaped), thickening and shortening of the base processes, and a decrease in their branching indicate the activation of microglia (30, 31). Most microglial cells in the hippocampal CA1 area of the control animals were characterized by small round or slightly elongated somas with thin, long, and moderately ramified processes, whereas LFD–, HFD–, and HFD+InsLB-fed ApoE–/– mice demonstrated morphological indications of microglia activation (Figures 4B,C). There were enlarged oval and, in some cases, rod-shaped somas along with both long ramified and short, poorly branched processes in the Iba-1-positive cells of LFD-fed animals. Microglia with big somas and branched processes of different lengths were detected in the hippocampus of HFD-fed mice. Mainly cells with small rounded or polar somas were detected in the HF+InsLB group; rod-shaped cells and signs of partial deramification were occasionally observed. The HF+WhLB group, in turn, harbored mostly morphologically resting microglia (small and predominantly rounded cell bodies and long and highly branched processes). Concerning the quantitative indicators, the number of Iba-1-positive cells per unit area was higher (*p* = 0.06) in LFD-fed ApoE–/– mice vs. those on standard chow (Figure 4F). The increase in the surface area of Iba-1-positive cells in LFD- and HFD-fed animals was substantial, by 25 ± 3% (*p* < 0.05 and *p* = 0.10), compared to the control group. Microglial hypertrophy in the HF+WhLB and HF+InsLB groups reduced by 25–30% relative to HF (*p* < 0.05) and LF (*p* < 0.01) (Figure 4G).

### Ultrastructural Changes in the Hippocampal CA1 Area

**Figure 5:**
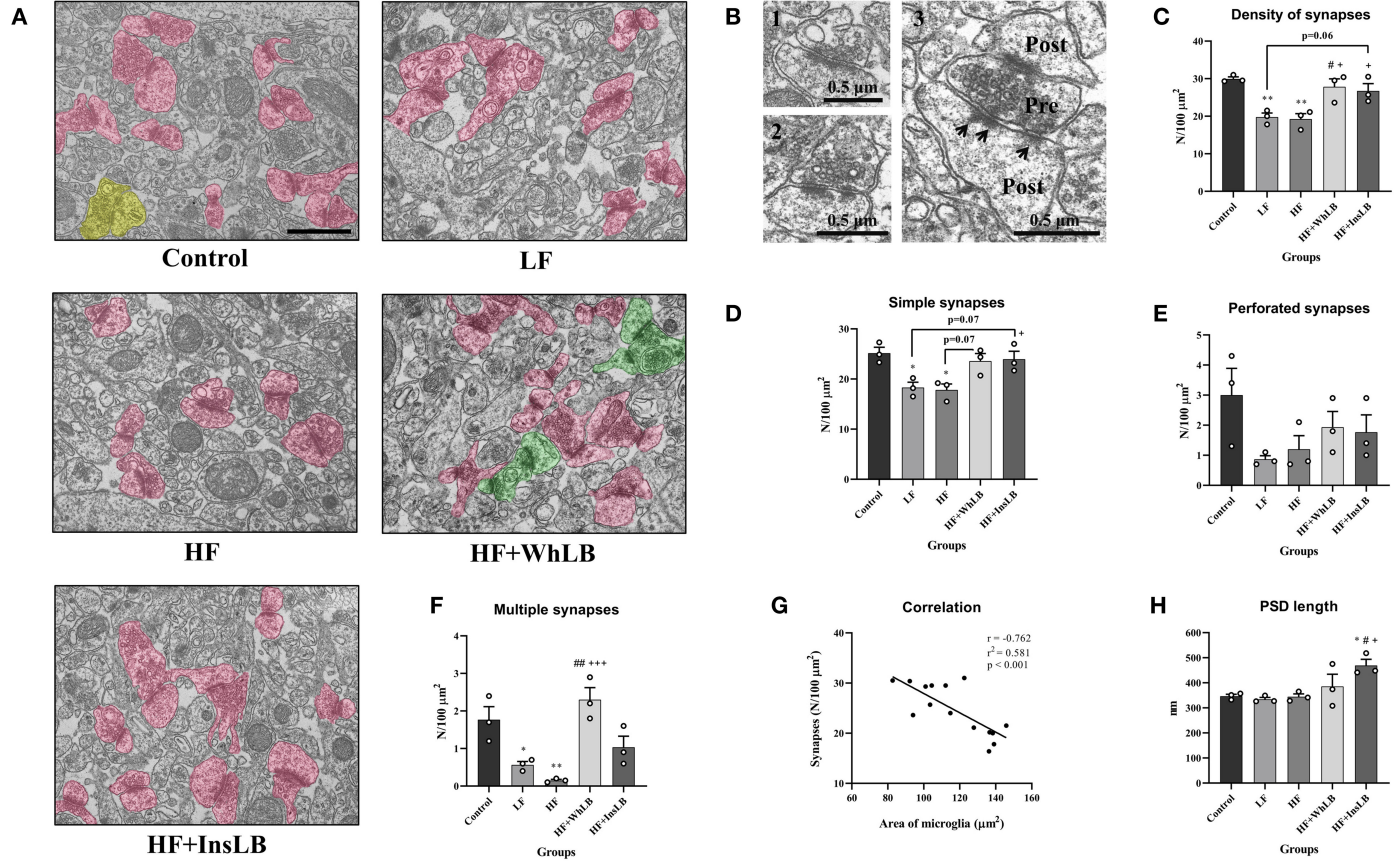
Electron microscopic analyses of the *stratum radiatum* in the hippocampal CA1 area of distinct experimental groups. **(A)** Electron micrographs of the neuropil ultrastructure. Different types of synaptic contacts are marked in various colors, as follows: simple—pink, perforated—yellow, multiple—green. **(B)** Representative images of (1) simple, (2) perforated, and (3) multiple synaptic types. Pre, presynaptic terminal; Post, postsynaptic terminal: Arrows point to the postsynaptic density. **(C)** The density of synapses and **(D)** the number of simple, **(E)** perforated, and **(F)** multiple synaptic contacts per 100 μm^2^ of hippocampal neuropil. **(G)** Pearson's correlative analysis between the area of microglial cells and synaptic density. **(H)** The average length of the postsynaptic density. Data are expressed as the Mean ± SEM (*n* = 3/group). Comparisons among groups were performed with the one-way ANOVA, followed by Turkey's *post hoc* test. *p < 0.05 and **p < 0.01 indicate significant differences vs. control; ^#^*p* < 0.05 and ^##^*p* < 0.01 indicate significant differences vs. LF; ^+^*p* < 0.05 and ^+++^*p* < 0.01 indicate significant differences vs. HF. The scale bar corresponds to 1 μm.

Ultrastructural analyses of the hippocampal CA1 area in different experimental groups of ApoE–/– mice (LF, HF, HF+WhLB, HF+InsLB) showed that the structures of their neuropil did not have critical morphological differences with those of the control animals (Figure 5A). However, some changes in the quantitative parameters of the neuropil were identified via morphometric analysis (Figures 5C–H). Control mice had 29.9 ± 0.5 synapses per 100 μm^2^ of *stratum radiatum* in the hippocampal CA1 area (Figure 5C). This parameter declined nearly 1.35 times in both the LF (*p* < 0.01) and HF (*p* < 0.01) groups compared with the control animals. Meanwhile, the inclusion of whole lingonberries and their insoluble fraction to an HFD led to an increase in synaptic density by 38 ± 3% (*p* < 0.05 for HF+WhLB and *p* = 0.06 for HF+InsLB) and 42 ± 3% (*p* < 0.05), compared to an LFD and an HFD without berry supplements, respectively. Given that the most prevalent synaptic type is the simple one, the differences in the number of simple synapses observed among experimental groups were very similar to the differences in overall synaptic density (Figure 5D). Of note, LFD- and HFD-fed mice demonstrated a dramatic decrease in the density of multiple synapses in the hippocampal CA1 area vs. control animals. The simultaneous ingestion of whole lingonberries and an HFD contributed to about a 15-fold rise in the number of multiple synapses (*p* < 0.001) compared to the intake of an HFD (Figure 5F). Interestingly, the hippocampal synaptic density had a strong negative correlation with the area of microglial cells (*r* = −0.762). According to the coefficient of determination (*r*^2^), we assume that microglia hypertrophy could cause the observed diminishing number of synapses with the likelihood of 58% (Figure 5G). In addition, there was a statistically significant increase in PSD length (by 35–40%) in mice on an HFD with InsLB supplementation (*p* < 0.05) relative to standard diet-, LFD-, and HFD-fed animals (Figure 5H).

### GFAP and NCAM in the Hippocampus

**Figure 6:**
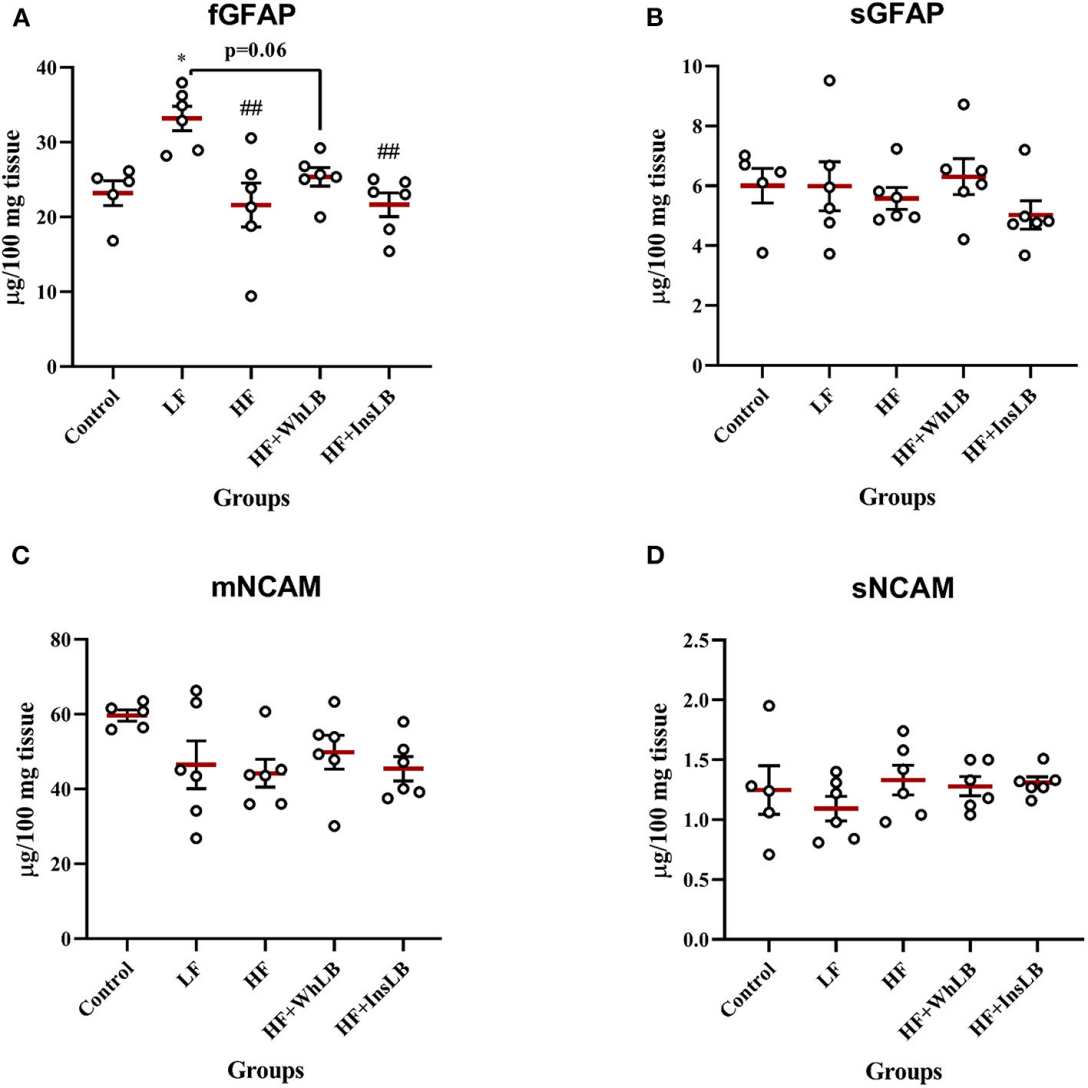
The levels of astrocyte- and neuron-specific proteins in the hippocampus of ApoE–/– mice. The concentrations of **(A)** the filamentous and **(B)** the soluble forms of GFAP, as well as **(C)** the membrane and **(D)** the soluble forms of NCAM are presented in the form of the scatter dot plots. Data are expressed as the Mean ± SEM (*n* = 5–6/group). Comparisons among groups were performed with the one-way ANOVA, followed by Turkey's *post hoc* test. *p < 0.05 indicates significant difference vs. control; ^##^*p* < 0.01 indicates significant differences vs. LF.

The highest level of the filamentous form of GFAP was observed in LFD-fed mice, accounting for a concentration that was 43, 54, 31, and 53% more than that in the control (*p* < 0.05), HF (*p* < 0.01), HF+WhLB (*p* = 0.06), and HF+InsLB (*p* < 0.01) groups, respectively (Figure 6A). Meanwhile, the analysis of the soluble form of GFAP did not reveal any differences among experimental mice (Figure 6B). In addition, neither investigated diet contributed to the modulation of NCAM expression, as is evident from our data of the concentrations of its membrane and soluble forms (Figures 6C,D).

## Discussion

It is generally believed that high-fat diets promote obesity and neuroinflammation and induce a cognitive decline in ApoE–/– mice (32, 33). Based on this understanding, we hypothesized that an HFD intake could induce the neuroinflammation and damage of brain tissues, particularly the hippocampus, eventually leading to cognitive impairments. Lingonberries and their insoluble fraction were anticipated to prevent, at least partly, the abnormalities mentioned above (20).

In the present study, comparing the experimental diets (standard chow, an LFD, an HFD, an HFD+WhLB, and an HFD+InsLB) did not reveal their influence on short-term spatial memory in young adult ApoE–/– mice. But surprisingly, animals on an LFD, unlike other rations, demonstrated a marked increase in the density of hyperchromic (damaged) pyramidal neurons and astroglial activation (namely, elevated levels of the filamentous form of GFAP, more significant astrocyte proliferation, and their hypertrophy) in the hippocampus. Reactive astrogliosis is generally understood to enhance neuroprotection and trophic support for neurons; however, it may further lead to emerging damage and neuronal loss through the production of pro-inflammatory cytokines and ion imbalance (34). Worth noting, the indicated morphological impairments in the LF group are consistent with our previous results derived from the 16S rRNA gene sequencing of cecal microbiota (unpublished data); in that study, LFD-fed mice harbored the highest *Firmicutes/Bacteroidetes* ratio and relatively abundant *Oscillospira* and *Desulfovibrio* among all experimental groups. Reportedly, a higher *Firmicutes/Bacteroidetes* ratio correlates with depression-like behavior, hippocampal astrogliosis, cognitive deficit, amyloid plaques load, obesity, and inflammation (35, 36), and an abundance of *Oscillospira* and *Desulfovibrio* is associated with systemic inflammation and impaired intestinal permeability (34, 37).

Morphological signs of astrogliosis, incidentally, were also observed in the HF+WhLB group. But, in our view, it had a different nature from that in LFD-fed mice. A possible cause of astrocyte activation in animals fed WhLB might be the high concentration of free benzoic acid in whole lingonberries (0.6–1.3 g/L, about 5% of all organic acids), which is water-soluble (appears in an InsLB fraction to a lesser extent) and ensures a low pH in the berries (pH 2.6–2.9) (38, 39). Per a previous report, 80 mg/kg bw/d of benzoic acid promotes sub-chronic oral toxicity in mice (40).

Interestingly, both LDF- and HFD-fed ApoE–/– mice had morphological signs of microglia activation (increase in the area of Iba-1-positive cells, enlargement of somas, alterations in the pattern of processes ramification in the two groups, and moderate microglia proliferation in the LF group), and a decline in hippocampal synaptic density alongside a reduction in the number of multiple synapses compared to ApoE–/– animals on a regular diet and an HFD with lingonberry supplements. Many recent publications confirm the relationship between microglia on one side and synaptic plasticity and neurogenesis on the other in some physiological or pathological conditions. For instance, the hypertrophy of Iba-1-positive cells in the hippocampal CA1 area in the mouse model of Alzheimer's disease has been associated with a substantial decline in dendritic spine density (41). Furthermore, HFD-induced obesity contributes to microglial reactivity in the hippocampus of C57BL/6J mice, as evidenced by an increase in the microglial soma area (without significant cell proliferation) along with the increased co-localization of Iba-1 and CD68 markers. In that case, microglial activation was involved in the loss of dendritic spines and synaptophysin-positive sites in the CA1 area and dentate gyrus due to microglial phagocytosis of synapses, higher expression of pro-inflammatory cytokines (TNF-α and IL-1β), and deterioration of neurogenesis in the dentate gyrus (42, 43).

Considering that we observed changes in hippocampal morphology not only between the mice fed an HFD and standard chow/an LFD, but also between the intake of an LFD and standard diet, we could assume that the lipid composition of the diets was not crucial in inducing the disturbances mentioned above. As seen from the composition of the investigated diets, the most substantive quantitative and qualitative differences were noted in dietary carbohydrates (Table 1; https://sdsdiets.com/wp-content/uploads/2021/02/rm1p-e-fg.pdf). The primary source of carbohydrates in the LFD and an HFD was corn starch (66 and 51 gm%, respectively). Additionally, both diets consisted of ~5.5 gm% of cellulose, insoluble dietary fiber. Notably, lingonberry-derived fiber and other berry components substituted some starch in an HFD (34 gm% for an HFD+WhLB and 37 gm% for an HFD+InsLB). Meanwhile, the standard RM-1 diet had 45 gm% of starch that originated from wheat, barley, wheat bran, and de-hulled extracted soya and 17 gm% of dietary fiber.

Generally, the more there is starch and low-molecular sugars and the less there is dietary fiber in a diet, the higher the glycemic index (GI) that diet has (44). Consequently, we can rank the diets used in the order of increasing GIs: an HFD+WhLB → an HFD+InsLB → standard chow → an HFD → an LFD. As shown before, feeding C57BL/6J mice with high GI-diet resulted in an increased blood glucose area under the curve (AUC) during OGTT and the development of insulin resistance (45). Similarly, in our experiment, ApoE–/– mice fed an LFD and HFD demonstrated enhanced AUCs by 16–24 and 10–17%, respectively, vs. eating standard chow and an HFD with lingonberries (unpublished data), indicating the risk of glucose metabolism abnormalities and insulin resistance in those groups.

Some authors have pointed out an unfavorable impact of excessive dietary starch on glucose metabolism. Maekawa et al. found that a high-starch diet (starch 74%kcal) considerably enhanced insulin secretion during OGTT and the value of HOMA-IR index in mice, a representative indication of insulin resistance (46). Patterson et al. in turn, revealed that a low-fat high-corn starch diet might cause more severe glucose intolerance in mice than a low-fat high-sucrose diet (47). However, not only total dietary starch content but its qualitative composition is relevant to glucose metabolic rate. Starch consists of two major components—amylopectin (branched and more digestible) and amylose (linear and less available for enzymatic hydrolysis). A high-amylopectin diet promotes a more significant production of postprandial blood glucose and the development of insulin resistance in rats compared to a high-amylose diet (48). Literature indicates that the amylose content in the primary plant sources of starch in the diets we applied are as follows: wheat (21.5–26.6 gm%), barley (25.8–29.8 gm%), and corn (20.9–25 gm%) (49–51). Thus, heterogeneity in starch composition between standard chow and an LFD/HFD might partly contribute to morphological changes detected in the hippocampus of ApoE–/– mice, but we tend to think that the overall level of this polysaccharide plays a more critical role.

Glucose imbalance and insulin resistance correlate positively with both structural and functional impairments in the hippocampus. Diet-induced hyperglycemia in middle-aged rats has been shown to lead to disturbances in spatial learning, reduction in LTP at Shaffer collaterals, and dendritic spine density in the CA1 area (52). Diabetic mice with chronic hyperglycemia exhibit enhanced blood-brain barrier (BBB) permeability, microglia activation (Iba-1 immunoreactivity, enlarged microglial somas), and elevated TNF-α and IL-6 production, along with the downregulation of the expression of two synaptic markers, spinophilin and synaptophysin (53, 54). Moreover, chronic high blood glucose level-induced activation of microglia and neuroinflammation exacerbates apoptosis in hippocampal pyramidal neurons (55).

As mentioned before, investigated diets also varied in amount and types of dietary fiber. Standard chow was more abundant in fiber, including hemicellulose, cellulose, pectin, and lignin, than an LFD/HFD with cellulose alone. Furthermore, various ingredients of a standard diet are not equal in the content of dietary fiber; for example, total dietary fiber account for about 13, 45, and 17 gm% of wheat grains, wheat bran, and barley grains, respectively (56, 57). Barley is worth emphasizing to be rich in β-glucan (~2–7 gm%). It has been shown that barley β-glucan reduces insulin resistance, serum glucose levels, and lipids in HFD-fed mice. Due to the modification in gut microbiota composition, barley provides an anti-inflammatory effect as well (58). Zhang et al. demonstrated that wheat fiber significantly diminishes the size of atherosclerotic plaques and, most importantly, inhibits the expression of pro-inflammatory factor NF-kB in the aorta of ApoE–/– mice (59). However, whether cereal fiber could modulate NF-kB expression in microglia, thereby impacting neuroinflammation in the hippocampus, merits detailed evaluation. In addition, dietary fiber supplements link with BBB integrity, hippocampal morphology, synaptogenesis, and cognitive function indirectly through the alteration of gut bacterial cenosis. Shi et al. revealed that an intake of microbiota-accessible carbohydrates (MAC) boosts the richness and α-diversity of fecal microbiota in obese mice, especially within the *Bacteroidetes* taxon, compared to a fiber-deficient diet. In the hippocampus, MAC supplementation to an HFD can attenuate impairments induced by fiber-deficient ration, such as a decrease in occludin levels (tight junctional protein), reactive astrogliosis and microgliosis, the upregulation of pro-inflammatory cytokines (TNF-α, IL-1β, IL-6), the engulfment of PSD95-positive sites by microglia, and the deterioration of the recognition memory (60, 61).

At the ultrastructural level, we revealed that rations with lingonberries and their insoluble fraction prevented the decline in synaptic density in the CA1 *stratum radiatum*, observed in HFD- and LFD-fed animals. In addition, lingonberry supplements altered the ratio of synaptic types, increasing the total number of multiple synapses. Beyond that, the consumption of the insoluble fraction of the berries supported the elongation of PSD. We see such reorganization as an indication of higher structural synaptic plasticity in the hippocampus. The formation of multiple synaptic contacts is thought to enhance the efficiency of synaptic transmission and is associated with several synaptogenic stimuli, such as brain damage, LTP, hippocampus-mediated training, estrogen use, and more (62). The PSD length increase, in its turn, is linked to the increased abundance in neurotransmitter receptors on a postsynaptic membrane and, as a result, more intensive neurotransmission (63). Interestingly, the enhanced hippocampal synaptic density in the HF+WhLB and HF+InsLB groups correlated with the decrease in microglial cellular area. Thus, in our study, microglial activation may serve as a mechanism of structural synaptic plasticity regulation. The synaptic improvements in the hippocampi of animals fed lingonberries and their insoluble fraction were also accompanied by a substantial rise in the relative abundance of *Akkermansia* in the cecum, as well as a decrease in *Mucispirillum*, unlike in the HF group (19). *Mucispirillum* is associated with gastrointestinal inflammation and age-related changes (64), whereas, *Akkermansia* normalizes lipid profile and blood glucose levels during obesity, attenuates Aβ pathology, restores neuronal development and synaptic plasticity, and provides anti-inflammatory effects (65–67).

The growth and maintenance of beneficial gut microbiota in ApoE–/– mice could be achieved through dietary fiber and phenolic compounds (e.g., quercetin, catechin, resveratrol, anthocyanins, and more), which are abundant in lingonberries (68). For example, a blackberry anthocyanin-rich extract has shown a neuroprotective effect in rats fed an HFD by modulating the gut microbiota and enhancing kynurenic acid production (a neuroprotective agent) (69). Combining quercetin with resveratrol restores HFD-induced intestinal microbiota dysbiosis in rats and increases the relative abundance of *Akkermansia muciniphila* (70).

Regarding the role of dietary fats, in our study, their excess in an HFD had a neutral effect on the hippocampal structure of ApoE–/– mice rather than a negative one. As illustrated in Table 1, all fats in standard chow and LFD constituted soybean oil, whereas an HFD contains not only soybean oil but also lard, which is rich in saturated (39–45%) and monounsaturated (42–45%) fatty acids (71, 72). Brain phospholipids have recently been shown to incorporate monounsaturated fatty acids more frequently than phospholipids in rodent heart, liver, and kidney (73). Thus, we do not exclude the potential impact of a lard-rich HFD on neuronal plasticity maintenance in young ApoE–/– mice. Besides, the 8-week feeding period might be insufficient for noticeable hippocampal and behavioral effects of an HFD to develop. Therefore, further investigations must be carried out to scrutinize this hypothesis.

Since different dietary exposures on animals with ApoE deficiency have being studied for more than a couple of decades, many divergent observations have accumulated. Their comparison is complicated by variations between experiments such as rodent's age and sex, composition of diets, duration of feeding, *etc*. For instance, Mulder et al. showed a dramatically increased BBB permeability in adult ApoE–/– mice with chronic HFD intake (74), whereas Bai et al. demonstrated that the consumption of an HFD elevated levels of GFAP, pro-inflammatory and pro-apoptotic factors in the cortex and hippocampus of 20-week-old male ApoE–/– mice (75). Another work suggests that 5-week feeding of ApoE-deficient rats with an HFD upregulated IL-1β and decreased the expression of occludin in the brain (signs of neuroinflammatory reaction), compared with an LFD (76). In contrast, there is evidence indicating the beneficial effect of a high-fat/high-cholesterol diet on the expression of BDNF and TrkB mRNAs in the hippocampus of 20-week-old ApoE–/– mice, which is related to improved synaptic plasticity (77). Meanwhile, our morphological and behavioral data are largely consistent with certain previously published papers. Crisby et al. also found microgliosis and no difference in the density of GFAP-positive astrocytes in the hippocampus of adult ApoE–/– mice fed an HFD, as compared to those on a standard diet (78). Other researchers, in turn, state that an HFD did not affect the spatial memory of middle-aged ApoE–/– females (4). In addition, the obtained results on the impact of lingonberries on the structural synaptic plasticity complement existing information concerning the ability of bioactive compounds from fruits and berries to correct the metabolic and neurological alterations in ApoE-deficient mice including reduction of the atherosclerotic lesion (20), enhancement of antioxidant protection (79), increasing the level of brain acetylcholine (80), and so on.

It is worth pointing out several limitations when interpreting and extrapolating the present results. Firstly, only males were examined in our study that constrains the spreading of the findings on the entire population of ApoE–/– mice. Secondly, due to the relatively young age of the investigated animals and thereby insufficiently marked neuromorphological and cognitive alterations, a favorable or adverse impact of the diets may not be fully manifested. Thirdly, additional research on wild-type mice appears to us would enable more comprehensively interpret the obtained data, as well as explores possible outcomes from the consumption of various dietary components, depending on ApoE genotype.

In summary, we revealed that ApoE–/– mice on an LFD were characterized by the hyperchromatosis of the pyramidal neurons and signs of neuroinflammation in the hippocampal CA1 area. By contrast, the consumption of an HFD did not substantially negatively affect the cellular structure of the hippocampus. However, both diets contributed to synaptic loss along with a shift in the number of multiple synapses and moderate microglia hypertrophy. Obtained results may be related not so much with the fat content of diets used but rather with the composition of carbohydrates. Our study demonstrates that reducing the starch amount and increasing dietary fiber in a ration is more favorable for maintaining the healthy hippocampal structure in young adult male ApoE–/– mice. The inclusion of lingonberries and their insoluble fraction to an HFD seems to provide a neuroprotective effect on the altered structural synaptic plasticity. Meanwhile, the above-mentioned morphological changes in the hippocampus of ApoE–/– mice did not escalate into severe spatial memory impairments.

## Data Availability Statement

The datasets generated for this study are available on request to the corresponding author.

## Ethics Statement

The animal study was reviewed and approved by the Local Ethical Committee for Animal Experiments at Lund University, Sweden.

## Author Contributions

GS, OP, FH, and NM designed the study. TK, IO, NM, and GU performed the experiment, sample collection, and behavioral testing. GU and DM carried out ELISA. DS, TK, IO, KS, and GS conducted morphological analyses and interpreted results. DS wrote the manuscript. All authors revised and approved the final version of the manuscript.

## Funding

This study was funded by the Direktör Albert Påhlsson Foundation Grant (No. 2019–169) and the Swedish Research Council FORMAS Grant (No. 2015–00877).

## Conflict of Interest

The authors declare that the research was conducted in the absence of any commercial or financial relationships that could be construed as a potential conflict of interest.

## Publisher's Note

All claims expressed in this article are solely those of the authors and do not necessarily represent those of their affiliated organizations, or those of the publisher, the editors and the reviewers. Any product that may be evaluated in this article, or claim that may be made by its manufacturer, is not guaranteed or endorsed by the publisher.
